# Exploring employees’ beliefs regarding the potential benefits of virtual worlds for group cohesion: gather town

**DOI:** 10.1007/s11042-022-14308-7

**Published:** 2022-12-23

**Authors:** Pedro R. Palos-Sanchez, Pedro Baena-Luna, Daniel Silva-O’Connor

**Affiliations:** 1 University of Sevilla, Ramon y Cajal Av, 1, 41018 Seville, Spain; 2 Open University of Catalunya, Rambla del Poblenou, 156, 08018 Barcelona, Spain

**Keywords:** Telework, Team cohesion, Virtual worlds, Gather town, Focus group

## Abstract

As a consequence of advances in Information and Communication Technologies, teleworking is becoming more and more common in organizations. These new ways of working create new challenges for companies such as team cohesion despite working in different locations. This article aims to analyze the effect of the use of so-called “virtual worlds” on the group cohesion of employees in organizations. The focus group methodology has made it possible to gather the beliefs and opinions of company employees about the use of these resources and tools. The results obtained show the positive effect of the use of virtual worlds on the cohesion of the teleworking team.

## Introduction

Even in recent years, we are witnessing an ever-increasing expansion and popularization of computer networks [28] and the application possibilities are increasing [31] in various application fields [52]. The most technologically native to the most traditional sectors are being influenced by these new conditions favored by the current possibilities provided by technology [29].

Recent developments arising from COVID-19 and the advance of Information and Communication Technologies (ICTs) have encouraged telework to take root in many companies around the world [1]. The need to avoid contagion using social distancing forced even those who did not believe in teleworking as a long-term arrangement to recruit the necessary technological and logistic means to support it [36].

Now that the lockdown is over, it seems that at least for the time being, telework is here to stay. According to [21], the percentage of companies whose employees teleworked regularly in the first trimester of 2021 for at least 30% of their shifts reached 12.16% for small companies, 18.91% for medium companies, and 25.72% for large companies. This, as we will discuss in further detail later, is quite the rise for a country that has traditionally favored face-to-face relationships at work. This change has had obvious consequences on how employee relationships and supervision are managed, as well as internal communication, and other important factors that influence company culture [19].

This article aims to provide a theoretical framework of quality teleworking and an approach to this problem from the point of view of social psychology, specifically, through the lens of group cohesion. A qualitative study has been carried out using focus groups to assess the perception of the use of virtual worlds as tools in the daily life of work teams. Specifically, the questions were aimed at assessing whether employees consider that these can promote cohesion compared to those currently in use, such as Zoom or Google Meets. The virtual world chosen for this task was Gather Town, which was presented to our participants before their discussion in case they have never used one before. Therefore, the research question of this paper is as follows:

- RQ. Do tools such as Gather Town favor group cohesion in work teams that work in a mixed way (face-to-face and/or telework) and/or only telework?

To achieve the proposed objective, this article is structured as follows. After this first introduction, the theoretical framework section addresses the fundamental concepts underlying the reality of telework and tools based on virtual worlds. Subsequently, in the methodology section, the key methodological aspects are presented when collecting, processing, and analyzing the data from which the results obtained will be shown. From the study and analysis of the information obtained, we will derive the discussion of these and the presentation of the most relevant conclusions of this work.

## Theoretical framework

### Telework in companies

The Spanish law 10/2021 on telecommuting [27] defines teleworking as remote work that is carried out through the exclusive or prevalent use of computers, telematics, and telecommunication means. According to Messenger [34], there have been three generations of telework. The first centers around the term “telecommuting”, meaning getting to work “virtually”, and that later would be used indistinctly with ‘telework’. The second would be difficult to distinguish from the first, calls this stage “The Mobile Office” because it would be associated with the progressive reduction in size, portability, and independence that electronic devices were gaining, which would later allow them to be taken to offices that were neither the worker’s home nor the offices of the employer. The third generation would come to include the possibility of teleworking not having a physical place associated with it, since it could be carried out, theoretically, anywhere, thanks to the great portability of devices such as cell phones or tablets.

In the European Union (EU), according to the European Commission [12] in the 10 years before the Covid-19 outbreak, its growth had been slow, but steady. Specifically, they note in their Science for Policy Brief [12] that only 5.4% of EU-27 workers were working from home in 2019, although the number of people occasionally working from home had increased from 5.2% in 2009 to 9% in 2019. Within the EU there was great variability between countries that allowed teleworking in these modalities. This variability also exists in between different professional sectors, with the IT industry at the forefront, understandably, along with others who because of the nature of their work are not required to physically go to their workplace. However, it is important to note that even within the sectors that did allow this occasional teleworking, there was also variability between countries, due to differences in legislation and so on.

Gschwind & Vargas [18] refer to this variability that can be found throughout the EU in a chapter dedicated to the effects of telework in Europe. Nordic countries, where telework in 2019 was already a more common reality, differed from southern and eastern Europe in the existence of a regulatory framework that allowed for a better balance between paid work and private life, work culture, and other aspects that made it easier to implement it. The greater demand for work-life balance in countries such as Germany was also considered a facilitator in this sense.

Numerous variables can influence an adequate adoption of telework. Teleworking can bring many advantages for both the employee and the company, but at the same time, it is a challenge since physical distance implies many changes compared to face-to-face work. Ultimately, it requires the company to adopt a flexible approach toward employee management and how the company culture is defined.

This flexibility is key for many reasons Pyöriä [40] points out that not everyone has personal or environmental conditions that are suitable for teleworking. Also, adaptations need to be made so that those who work remotely do not lose the opportunity to bond with their colleagues. Flexibility also means being open to the idea of teleworking from the start, and resistance to teleworking by management can be a problem when such opposition is based on fear or personal beliefs rather than hard evidence.

Teleworking allows the employee to have more autonomy and more flexibility in the organization of his or her work. However, surveys at the European level taken into account by Gschwind and Vargas [18] state that teleworkers invest more over time than their colleagues who work face-to-face. Additionally, what has been gained in terms of conciliation thanks to flexibility, can be lost in quality of life as a result of the intrusion of professional life into private life [11]. All this implies that the company must take the necessary measures to prevent these psychosocial risks. Another important aspect of teleworking, the key to maintaining a positive climate in the company, is the equity between workers in the different modalities.

Miglioretti et al. [35] proposed a study in which they made a distinction between high and low-quality telework. High-quality teleworkers would possess an agile work environment both inside and outside the office, time flexibility in terms of the organization of their work, and virtual leadership, measured by the clarity of their work objectives. They posed a series of hypotheses based on Bakker & Demerouti [4]: that workers in the high-quality telework condition would have more resources (measured in labor control, quality of their relationships and support), and so it was, except for social relationships, in which they found that even these workers showed a loss of compared to their colleagues in face-to-face modalities. They also tested the hypothesis that teleworkers tended to do more overtime work than face-to-face workers, but that with those in high-quality telework conditions these differences would not be found, and indeed, they were not. They also found greater engagement and balance between personal and family life in high-quality teleworkers than in low-quality and face-to-face workers.

In general, workplaces with intensive use of ICTs share a new health risk known as technostress. Salanova [44] considers three subtypes to facilitate the understanding of the term: *techno anxiety* (high levels of unpleasant psychophysiological activation due to the present or future use of ICTs), *techno fatigue* (fatigue and mental exhaustion related to the use of ICTs, as well as feelings of ineffectiveness about their use) and finally, *technoaddictionue* (the compulsion to always use ICTs and in all places).

### Virtual worlds and virtual teams

According to Aten [3], virtual worlds began to appear in the 90s, but the interest in them for this difference to entertainment starts in the year 2000 when Linden Labs launched Second Life. Second Life honors its name in the sense that it acts as a parallel world in which even economic transactions are carried out using the world’s currency. Users can create avatars of themselves, invest in real estate and participate in communities on the platform in the same way as they would in real life. Since then, several alternatives have sprung up and their use has covered everything from education, with university projects, to marketing with the creation of a virtual island by Coca-Cola [3].

In the case of virtual teams, they could be defined as groups of people who work together from different geographic locations, and that is sometimes also in different time zones using ICTs to complete their tasks [17]. Although for this study not all of our participants will match this exact description since employees who telework in hybrid modalities are also being considered.

### Group cohesion

Group cohesion is a concept that has been studied for several years now. An initial definition was purposed by Festinger et al. [13] as the forces that keep a group together. Cohesion is a concept that is at the heart of many key topics for many disciplines, but there has been great difficulty in finding a common definition that everybody can use for their research. Carron & Brawley [8] highlight some of these problems, reminding researchers that the conceptual model they had developed for sports teams must be adapted for its use in other contexts.

Forsyth [14] proposed that a possible solution may lie in the consideration of cohesion as a unitary concept, which at the same time acknowledges its precursory conditions, which for many authors have been in themselves elements that have served to evaluate the degree of cohesion in a group [15, 39]. Salas et al. [45] tackled how cohesion has been conceptualized and measured and its relationship with performance. They concluded that a multidimensional definition of cohesion is best, which means considering at least two dimensions depending on whether what motivates the individual is to fulfill group objectives and tasks, in which case one would speak of task cohesion, or whether it is to maintain interpersonal relationships with the rest of the members, where one would speak of social cohesion.

Although the idea of using virtual worlds for work or business purposes is not new, it never really became a mainstream practice. But only recently Facebook (now Meta) announce their plans regarding the companies’ business in virtual worlds and virtual reality [43] and since then so have many others like Microsoft or Nvidia. Group cohesion has been found to correlate with several indicators that are interesting for companies. One of the most important ones is, of course, group performance. Cohesion in education and the business environment should be differentiated in meta-analyses. As pointed out by Castaño et al. [9] this is because the objectives of workgroups in business tend to involve groups for longer periods, and their environment, in general, is different concerning the educational context.

Another key indicator would be job satisfaction, although the amount of evidence in this direction has not been as bountiful. [42] built on the literature surrounding group cohesion and job satisfaction by exploring the relationship between group cohesion and collective efficacy and life satisfaction, through job satisfaction. They found that cohesion has a stronger relationship with life satisfaction than collective efficacy, both when it was mediated by job satisfaction and when it was not.

### Group cohesion in virtual worlds

Many of the studies that have been conducted on cohesion in virtual environments come from the educational sector in the investigation of collaborative tasks by students.

Thus, Altebarmakian & Alterman [2] studied a group of students working remotely on collaborative tasks and at different moments in time and they concluded that the degree to which group cohesion developed depended significantly on the design of the learning space. In a related field, Bozanta et al. [7] sought to measure the perception of cohesion within a team in a Multi-User Virtual Environment (MUVE). They found that when users’ expectations about the game interface were met, their perception of cohesion increased and also, and they found a positive relationship between attitudes and cohesion; when participants liked the game and were entertained, they scored higher on cohesion.

Paul et al. [39] focus on global virtual groups that are divided into smaller subgroups, for example, those located in different geographical areas. They explored the role that cohesion and trust play in how effective coordination is within subgroups by selecting two samples of students from different countries (in this case, the US and India). They designed a model to study how cohesion and trust impacted coordination between both groups of students and found that they reinforced each other in a loop that could benefit group performance in general, highlighting the key role both play in groups working efficiently.

### Gather town

Gather Town is a video chat platform that uses 2D maps and avatars so that users can interact with each other in a way that better resembles reality. It launched in May 2020, and it is considered one of the many metaverses that are available today. The platform’s use is not restricted to a specific type of activity, such as business, but can also be used for informal meetings, educational purposes, or events.

Users interact with their virtual surroundings (called Spaces) via their avatars, both of which they can customize to fit their preferences and needs. Spaces act as real physical locations, in a sense, as when a user moves closer to another with their avatar, they gradually become able to hear and see them. There are also what they call private spaces inside the general Space users make use of. Visually, one of these could be, a coffee table with a few chairs, for example, sitting inside a bigger room, where only those who share this smaller space can hear and see each other, although their avatars can be seen from the outside.

There are also other useful features on the platform like file sharing, screen sharing, embedding video from popular platforms such as YouTube or Twitch, broadcasting to the whole room, and even integrated games.

Because the platform was launched in 2020, not many studies have been found that have used it for research, most of which are in the field of education. If there is one thing both scientific gatherings and online teaching have in common when turning to Gather Town as an alternative to traditional video calls, is that it seems like it offers a more interactive experience. Perhaps this perception of higher interaction could lead to higher levels of cohesiveness within a group that uses this kind of platform regularly? Unfortunately, the answer to this question lies beyond what information this study can provide but know what attitudes participants have towards these platforms and their potential, is a necessary start.

### Previous work on gather town

Because the platform was launched in 2020, not many studies have been found that have used it for research, most of which are in the field of education.

McClure & Williams [33], for example, investigated its use in higher education teaching statistics using pre-recorded videos, demonstrators, and training 38 students in practical skills using this platform. The results from this mixed-method study were limited by the fact that not all students could submit their responses by the time it was published, but it does offer some interesting insight into the user experience in a training context. Participants, both students, and teachers considered it was an interesting alternative to static video tools such as Zoom, especially regarding informal communication.

Standl et al. [47] performed another mixed-method study with 9 pre-service teachers in computer science and 7 computer science majors, to assess students experience with the tool through a post-questionnaire. The quantitative side of this study used the ratings given by the pre-teachers regarding the feeling of community, quality of group work, and learning achievement, while the qualitative side examined opinions about the quality of the work.

In general, the results from Standl et al. [47] showed that students perceived the same degree of learning as in face-to-face settings from the quantitative part of the research, and that synchronous work and active participation were perceived as better than face-to-face classes.

Bekeš et al. [5] consider this application an example and a method to increase student participation and create a more engaging environment, as the social components of an online event are enhanced. Other authors who also made comparisons with the Zoom tool and with students expressed the same opinion [26]. Sriworapong, et al. [46] investigated students’ engagement, enjoyment, and sociability in Virtual Reality-Based Systems. In the same vein, Lugo et al. [32] researched this tool to enhance a virtual summer camp experience in industrial engineering.

For Zhao & McClure [53] this tool has proven to have great potential for language teaching and is suitable for enhancing second language teaching, as it is a gamified video conferencing platform that revolutionizes the traditional static video conferencing experience for language learners. Offering a 2D map with avatar-based game features using proximity-based videoconferencing functionality, increases participation [37] and promotes interaction that could be compared to real-life conditions.

It seems Gather Town could offer the perception of higher interaction which could, in turn, lead to higher levels of cohesiveness within a group that uses this kind of platform regularly. We hope this study helps in shedding some light on this topic.

## Methodology

Focus groups were chosen to obtain the necessary information for this qualitative research. This method relies on groups of participants that can be recruited in various ways. In this case, because it is more probable that these kinds of tools will be used in the IT sector, participants were recruited from technological firms in Spain.

**Fig. 1:**
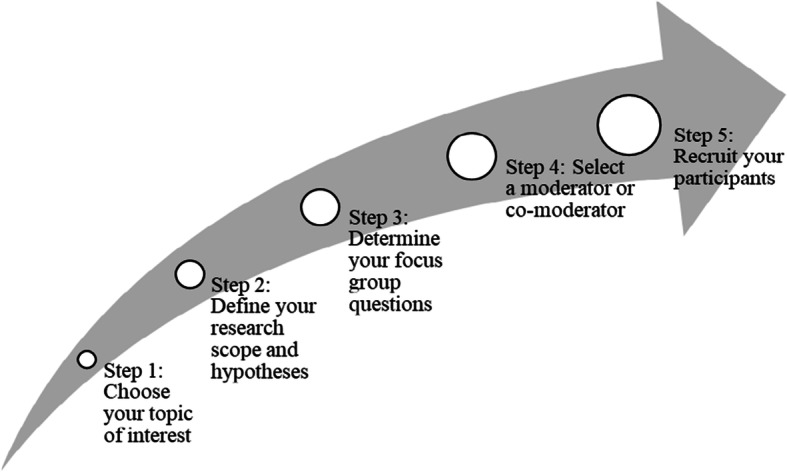
Flowchart of the study: focus group

The idea behind this study was to research employees’ ideas regarding the use of virtual worlds and the impact they might have on group cohesion within teams. Virtual worlds, however, are currently not used in most companies as tools for everyday work. They seem to be an emerging trend that could lead to them being used in this way, but that is yet to be decided (Fig. 1).

**Fig. 2:**
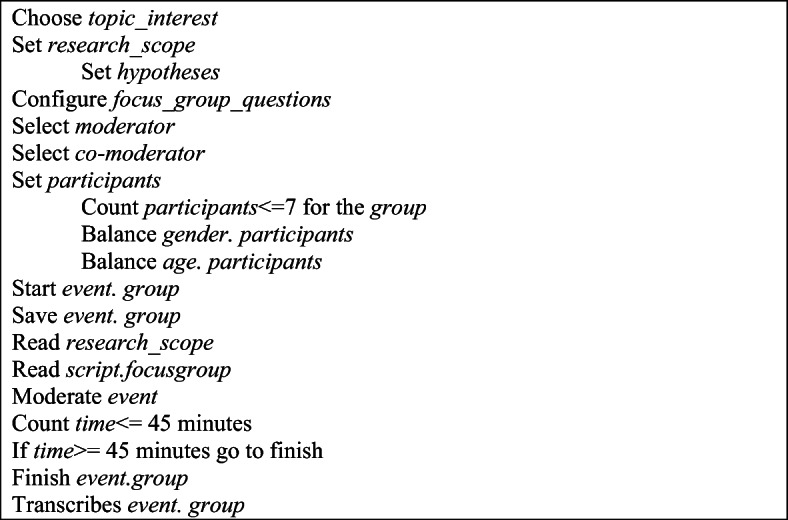
The algorithm in steps of focus group event

The algorithm in steps format about the research process is presented in Fig. 2.

### ¿Why focus groups?

One of the main advantages of using focus groups is, according to Litosseliti [30], that they present a more natural environment for participants to express their views, and that they allow for the inclusion of several views on a given subject.

The idea behind this study was to research employees’ ideas regarding the use of virtual worlds and the impact they might have on group cohesion within teams. Virtual worlds, however, are currently not used in most companies as tools for everyday work. They seem to be an emerging trend that could lead to them being used in this way, but that is yet to be decided.

For this reason, focus groups were considered the best option available to answer our research questions. They would be able to provide much more information and possibly suggest new paths worth investigating, which is ideal for an exploratory study.

### Participants

The only condition that was mandatory to participate was to be a technological firm employee. These kinds of employees work in the tech sector, which also gives them insight into technology-related news, as well as a realistic view of the pace at which more novel tech developments take over the previous one [49]. This means they probably have an advantageous standpoint from which to consider whether these tools could have a place in our workplaces in the future, ad if they would help our teams stay together.

All participants in this study were informed about the institutional review boards (IRB) and expressed their approval of the IRBs. All studies involving human subjects must first demonstrate compliance with the specific regulatory criteria for IRBs [6].

Employees were scouted in two consecutive waves. Given the research topic, a convenience sampling method was used, but with all working members of the same company. For this purpose, an email was sent to them asking for their collaboration.

The most accessible people were those who participated in the first group. As far as possible, an attempt was made to apply the recommendations of stratified sampling according to age and gender.

During the first one, a formal communication was sent to 25 employees that were randomly chosen from a mixture of departments within the company, with the idea of forming 5 groups with 5 participants each. The initial selection was also gendered balanced, and an extra 10 participants were chosen, although not contacted, in case any of the initial 25 did not want to participate, or in case there were any cancellations.

One week later, participants who had not answered were contacted through the Slack application and asked for a response. Most of them were recruited through this follow-up, which was expected. A few had to be added from the “emergency pool” since not all who had been initially contacted answered.

As participants confirmed and signed the necessary data and image rights agreement, they were grouped into 5 groups of 4 employees each. The decision of reducing the final number from 5 to 4 was due to several reasons: the time needed to confirm each participant, the deadline for this project, and that 4 participants would still allow for a decent amount of information to be recorded with more time for each speaker.

As for group composition, two of them were unbalanced by gender due to organizational issues, leaving them in one case with 3 men and 1 woman, and 3 women and one man in the other. The other three groups were balanced. One of the groups was made up of interns from different areas, they were grouped because they had to complete the activity during their working day, which in their case was the morning. Contracted employees, on the other hand, tended to have more free time in the afternoons.

### Materials

Participants were only required to have a computer with a camera and a microphone. The camera is not strictly necessary for the focus groups themselves, as only the transcript of the conversation will be analyzed on this occasion.

For the research itself, the free version of Gather Town was used. To record the activity and focus group, OBS, which is a screen and audio recording software was chosen, since it is available for free and produces good-quality video and audio files.

Sony Vegas was used to separate audio and video files, as well as to render the initial recording into an audio file, and Word online was used to create an initial transcription of the recordings.

To design questions that could help participants give answers that could be relevant to the concept of cohesion, We followed some of the guidelines mentioned in [45]. The first one, regarding the definition and measurement of cohesion, refers to asking questions that could give participants a chance to give their opinions about both the potential of these platforms to enable task and social cohesion.

Other guidelines that were used to ensure that participants were able to understand and answer the questions with the information we needed, were those from [24]. Specifically, questions were designed to act as a funnel, from more general questions that introduced the topic of virtual worlds and Gather Town, then questions about the meaning of unity in groups for participants, and lastly our key questions about cohesion.

We tried to always use open questions and simplified or explained terminology that might not be familiar to participants, such as group cohesion itself.

Additionally, although many participants will likely know each other from working in the same physical space or attending company activities, it is important to note that most individuals from these focus groups do not work as a team on a day-to-day basis, meaning that although they completed a task on Gather Town together as a group, they cannot be considered a mature group. To test the questions and general procedure, a beta focus group was done before the 5 formal focus groups where questions were further improved.

The idea behind this beta group was mainly to analyze the following: were our questions clear enough? Were there any terms that induced confusion or that needed to be clarified? What were the approximate length of the activity and the complete session? What directions did the group need to give enough information on a particular topic? Did they need any additional cues?

Thanks to this beta group, we simplified the language that was used a bit more and gave a brief introduction about cohesion, so they would have it in mind during the session (although we used the term “united” to make it more understandable). We also explained what we meant by usability, as more than one interpretation of the word came up in the beta group.

### Procedure

The focus groups were conducted in five sessions. There were two parts to each session, which were briefly detailed to them again via e-mail on the day the focus groups were done. An attachment explaining the basics of what was going to be explained in Gather Town was also sent to them with this e-mail.

The first part was an activity that was meant to act as a prompt for participants to later be able to speak after having had an experience with a virtual world. Gather Town was the application chosen for this task, which lasted approximately 30–45 minutes depending on the group. During the first 10 minutes or so, they were introduced to the platform where the basics of moving around, interacting with each other, and customizing spaces were explained.

**Fig. 3:**
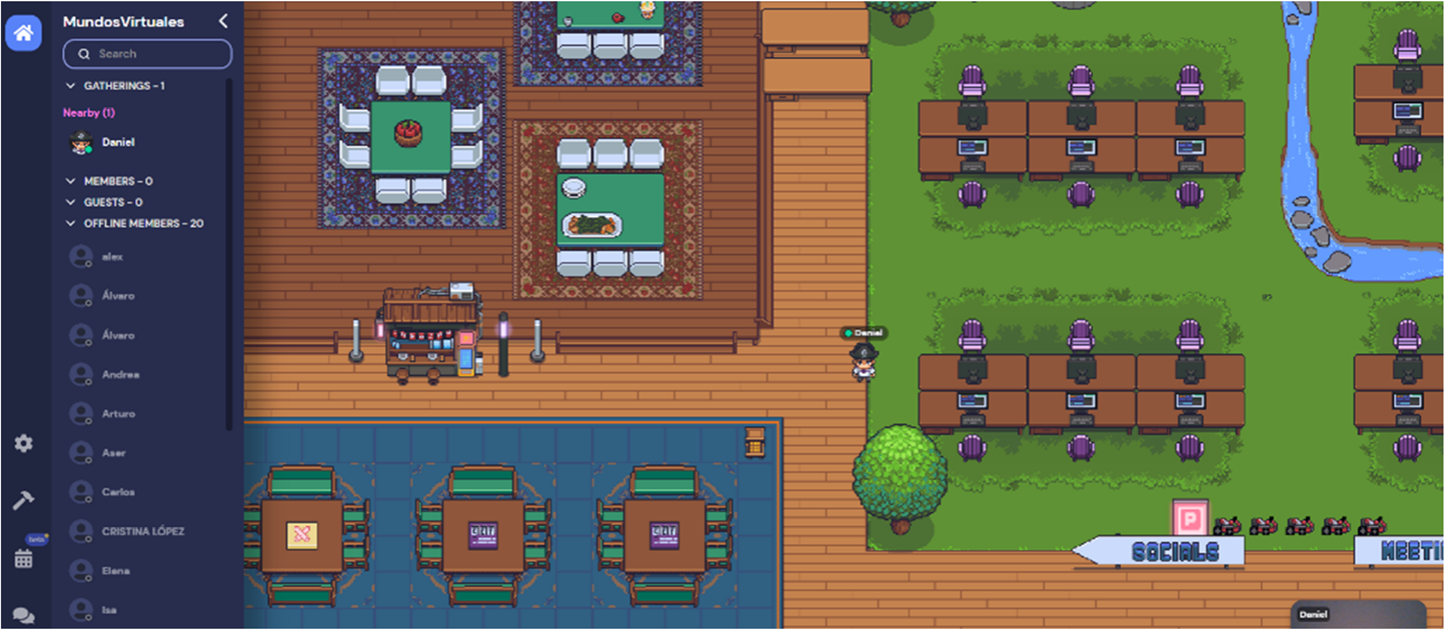
Premade template. Source: Gather Town application

A premade template provided by Gather Town was used for this (Fig. 3).

**Fig. 4:**
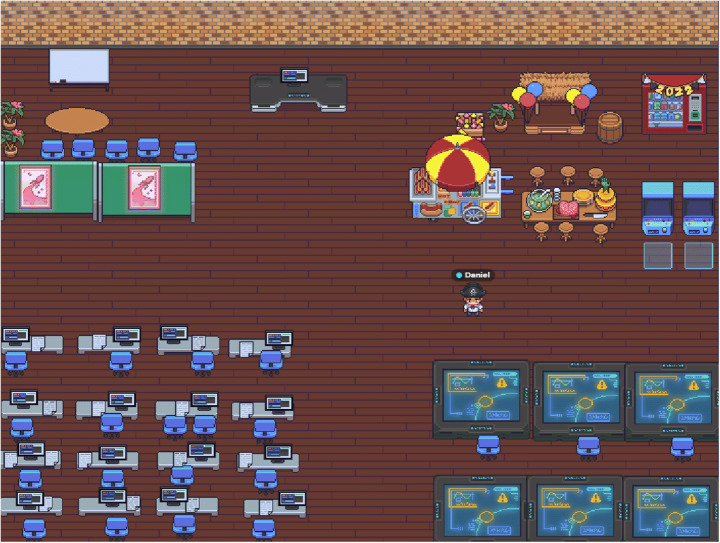
Completed activity room. Source: Gather Town application

After this, we moved to an empty room where they had to complete a collaborative task, which was customized to become their new company office (Fig. 4).

The collaborative task was meant to give them a chance to experience what working on a specific task as a team using virtual worlds would be like. They were given a flexible time cap of 15 to 20 minutes, after which they were asked to stop, and told the actual focus group would begin.

The focus groups lasted between 30 and 40 minutes depending on the group and the follow-up questions that were prompted by the conversation.

All of the research that has been presented here was done by the author, which means the same person wrote the questions, guided participants through a presentation of Gather Town, accompanied them throughout the collaborative task, and immediately after moderated the focus group. Everything was video recorded, and the audio file from the focus groups was used to extract the transcriptions.

The analysis and discussion of results, which includes coding data, was also done by the researcher, which has implications that will be discussed at the end of the study when talking about its strengths and weaknesses.

The method chosen to organize and draw conclusions from this data is Thematic Analysis as has been described by [25]. Table 1 shows how these steps have been adapted for the analysis of these focus groups.

**Table 1:** Steps analysis focus group

Steps as described by [25]	Steps were taken for this focus group
Initial work with the text: highlighting relevant information, composing memos	This was done during the transcription process, where the data that was unrelated to the topic or one related to it was discarded
Developing main topical categories	These categories correspond to the information the focus group questions aspired to obtain, such as, had participants had any previous experience with virtual worlds previously.
First coding process: code the available information using the available data	For this purpose, each answer was paraphrased and classified as a response to the different questions posed to the participants.
Compile all of the passages assigned to the main categories	After this, answers from the 5 different groups were compared.
Determine sub-categories	From these answers, new sub-categories were determined.
Second coding process: code all of the data using the elaborate category system	Codes were modified to fit the new system
Category-based analysis and presentation of results	Results were presented for each sub-category

During step one, each transcription was corrected to eliminate words that had been interpreted incorrectly by Word’s automatic transcription tool, which might lead to errors when using certain tools in MAXQDA.

This transcription could be considered to be something in between an abridged transcription and a complete transcription according to work by Krueger [23]. This means that transcriptions were adapted to what the speaker said as most much as possible, but without the strictness of a Verbatim transcription. Interventions made by the researcher were not corrected, as they have not been considered for this study except for the contextual information that might have been given for certain questions. Keeping the research question in mind: employee perceptions and beliefs regarding the use of virtual worlds to better group cohesion.

## Results

### Results per category and sub-category

#### Previous experience with virtual worlds

None of the participants had had any with virtual worlds as such, although they mentioned a few games where they would have had to move using avatars in a virtual setting that is similar.

#### User experience

The second question was about user experience. This question gave way to several new codes because although the meaning of “usability” was clarified. Nevertheless, these comments were interesting to the topic of research as they could help understand why they might consider Gather Town or other virtual worlds to be useful tools to generate cohesion. For this reason, a series of extra sub-categories were created under “usability”. When a comment was coded as “usability”, this meant that it spoke about the user’s experience with the platform. When the participant gave additional information, this was considered a sub-category within “usability”. Thus, the following categories were created (Table 2).

**Table 2:** Categories and sub-categories of usability

Category name	Number of coded segments
Usability	43
Usability \ Individual differences	4
Usability\ advantages	5
Usability\ advantages\ contextual information	12
Usability\ advantages Fun\ Entertainment	5
Usability\ Disadvantages	2
Usability \ Disadvantages \ Not much new	2
Usability\ Disadvantages\ Not suitable for large groups	3
Usability\ Disadvantages \ Other	3
Usability\ Disadvantages\ Distraction	6
Usability\ Potential work uses\ Inputs	6
Usability\ Ease of implementation	20
Usability Ease of implementation Complex implementation	8

“Usability” was the master code where any responses that related to the usability of the platform were included. Some of the responses here overlap with secondary codes such as implementation if someone talked about how, for example, they felt that having 200 users active in Gather Town could impact their user experience. This is why there were more answers in this master category than in other smaller ones, and why the individual subcategories do not add up to the master category.

The next section within usability was advantages, where most participants made two kinds of comments: on one hand, they thought it was a fun experience, and on the other, they thought the application gave users more context information from other users, which was something they felt was often lost when teleworking.

These were quite varied, some of them commenting on how they felt some options could be clear, how the application could be a distraction, or if it would be a good choice if there were too many users. Before this, however, we have a category for things that Gather could bring to the table, or how usable it would be for these specific employees.

The last category in this section was about how easy it would be to use a virtual world such as Gather Town. Generally speaking, participants thought that although it could be taken advantage of for certain uses, its everyday use would be complicated.

Please find an example for this section below.

**Table:** 

Group	Comments
3	“A more basic platform would be much more usable, wouldn’t it? I’m going to this place, but you don’t have to take a dummy and move it. Make it more like I’m going to this place, but without having to drag and drop an avatar, right, but you know what I mean? Maybe a screen. With maybe a mouse click in the room instead of moving a doll in the room, for example.
3	“If, in the end, if you use it well and so on, imagine if there were like working stations, you arrive, you stay here and you’re there all day, you get to work. Better than just sharing your status: Busy, in that sense, you do have a little bit of a vision of what each one is doing. For example, if this person is in a meeting, I better not bother him or whatever”.

#### The meaning of being united

The concept of cohesion can be a bit complex if presented out of the blue. For this reason, it was slowly introduced by using a few introductory questions, the first one being, ¿What does it mean when we say that a group is united? It is not an easy question to answer, and most participants did so by listing what they considered a group must have for it to be this way.

When comparing results by groups, having good communication and common objectives were mentioned several times in all five of them. After that, other different groups gave more specific insight into certain aspects, such as prioritizing the groups’ interests over individual gain.

Two additional sub-categories were created for the answers that came up in “the meaning of being united. The first one was named tools, and it was where all of the mentions of how different tools or programs could help or become a problem regarding unity came up.

Two main discussions came up in this section: in group 3, they discussed the importance of having your camera on while participating in meetings, and in group 2 they talked extensively about how the different software tools they used in their day-to-day affected communication and their general interaction. They commented on a few interesting points such as how there are different levels of formality in the language that is used in each tool (emails, for example, would tend to be written in a much more formal tone), and how this, in turn, influenced how comfortable they were communicating in different contexts. Another interesting line of discussion was how tools like “Slack” helped them interpret their colleagues’ answers, or lack of responses, by providing information about their availability.

The last set of answers to the questions about groups being united, was categorized as “prerequisites” of unity, or “facilitators” as they were answers to the follow-up question “What do you consider necessary for a team to be united?”

Group 1 did not give much more information for this question other than what they commented about what it meant for them for a group to be united. The rest of the 4 groups all coincided in that what they considered most important was their co-worker’s attitudes toward the group. Group 4 is defined in terms of predisposition to the word as a team. Group 3 talked about the importance of having “the right attitude” in terms of being involved in colleagues’ work.

Group 5 also talked about “pre-disposition”, and the person below mentioned the volitional element of this, which was also, to a certain extent, hinted upon in other groups as well. Although all groups agree on the attitudinal factor, some participants did talk about it as if it was more possible to influence through, for example, routines, as did group 5 when talking about using breaks to connect with their colleagues through Gather Town. Group 3 on the other hand, tended to regard these individual differences as part of the personality, which comes across as more consistent over time.

Only one person in Group 2 mentioned the importance of having a manager. They gave special importance to how the different tools they had at work could also be enablers of “unity” or cohesion.

Please find another example of this section below.

**Table:** 

Group	Comments
4	“Sure, in the end, if the team is united, communication is more fluid, and all those kinds of problems don’t happen because it’s much easier to communicate with someone you trust.”

#### The effects of physical distance on being united

This category was used for all comments that answered the question “Would you say physical distance can affect a team being united?” When needed, a follow-up question regarding their experience working during lockdown was asked, as well as if they considered this period working remotely had affected team cohesion.

Another 2 sub-categories were added later, one of them called “protective measures” for when participants proposed or talked about things, they or the company had done to encourage group cohesion when working remotely, and the other “specific aspects concerned”, when the participant specified the element of their working life distance was influencing.

Generally speaking, most participants did consider physical distance to have some kind of effect on groups, but discussions went differently in each group.

In Group 1 for example, they said they didn’t believe it affected group cohesion, but that it affected other things, such as how information was shared, how easy it was to ask for help from colleagues, or general socialization. However, after three contributions in this direction, the last participant commented that she thought all of those things would end up affecting cohesion, and they agreed.

Two members of Group 2 and Group 4 are interesting to compare because they both started working remotely, but with very different experiences.

Group 5 for example, commented on the effects they felt distance had had on group projects they had as students with large numbers of participants. Some of them commented that they felt large workgroups, of 20 people or so, were more affected by distance, especially in terms of how much work each person did.

**Table:** 

Group	Comments
2	“I joined just after the pandemic, and I have colleagues that I don’t know in person, but hey, look, with the four times that we have spoken and even virtually, you end up talking about your weekends and so on”
4	“I also went in post-pandemic, and I handled it terribly. But terrible, Because as I said, I didn’t know how the (other) one was, what the reactions of the rest were like, what the opinions of the rest were like, and so on”

#### Task cohesion and virtual worlds

Here are the answers to “How beneficial (or not) do you think the use of Gather Town or other virtual worlds could be for teams that need to work on a specific task?” No further sub-categories were created here because they weren’t considered necessary.

Many participants were unsure about how Gather Town or other virtual worlds could be implemented in their line of work for performing specific tasks, although they did come up with a few scenarios where it could be useful.

**Table:** 

Group	Comments
2	“For my work, it might not be worth it, but well, I do know other projects where people are connected all day long to Meets, so, in the end, you also get tired of having one person all day long, hey, and this, hey, and this? Well, it could be interesting when I’m in the same call with colleagues that are talking about things not relevant to my work, so it would not be bad if each one is in a room and if you want to talk to me, you come to my room. In other words, it would not seem bad to me in that sense.”
1	“Imagine that we achieve a virtual environment where you can share the code you send it to me via Slack”

#### Social cohesion and virtual worlds

Here are answers to the question “How beneficial (or not) do you think the use of Gather Town or other virtual worlds could be for teams in terms of their interpersonal relationships?” As with the previous category, no subcategories were created in this case either.

Participants were, in general, more positive in their views regarding the potential of Gather Town to benefit relationships between them. The main reason for this seemed to be that they thought it was fun.

**Table:** 

Group	Comments
5	“Well, for example, I see this as more useful because of what I was telling you before, for breaks, then we can stay in a room, and we are talking more than about just work”

#### The future of virtual worlds and other virtual experiences

The objective of this question was to explore thoughts on the feasibility of virtual worlds being used in the workplace, to talk about how they might evolve in the future, and if they would prefer them over the tools that they use in the company at the moment.

For group one, even though they concluded they would rather continue using Teams or Meets over virtual worlds or other more immersive alternatives like virtual spaces that used headsets, the key element seemed to be that they needed the tool to show their colleagues’ faces.

Group 2 did not give extensive feedback on this question and commented that they saw these tools as complementary to face-to-face meetings. One of the participants also said she would not feel as comfortable using it with her boss, presumably because it would stop her from showing a more “informal” side of herself.

Group 3 also tended to compare the use of these technologies with face-to-face meetings, even though it had been explained that their use was never intended to substitute real-life interactions.

Group 4 started an interesting discussion on how people might embrace the future of these technologies. They talked about how age and the habits the current population have using technology and social media might influence acceptance of future technologies like virtual worlds, or metaverses.

Group 5 did not expand or give enough information on this section, as most of their comments were more related to how Gather Town could be used in the office, rather than what the future of work would be, and their input has been considered in previously sections.

#### Survey

Finally, based on previous work on Gather Town [5] and uses and gratification theory (U&G) [22, 41], a short questionnaire of eight questions or items on Functionality, Entertainment and Satisfaction was constructed.

Based on a Likert 5 scale ranging from Strongly Agree-5 to Strongly Disagree-1 to Neither Agree nor Disagree-3, twenty-five questionnaires were collected from the focus group participants.

The results shown in Table 3 present the obtained means and standard deviations.

**Table 3:** Questionnaire results

Reasons	Item	Based Authors	average	standard deviation
Functionality	[FUN1] Interaction and learning with peers in non-face-to-face classroom activities would be enhanced by using applications such as Gather Town	Bekeš et al. [5]	3.80	0.84
Entertainment	[ENT1] Gather Town could be entertaining for me [ENT2] Gather Town looks like fun to use [ENT3] With Gather Town I stimulate my mind	Choi et al. [10] Katz et al. [22] Rauschnabel et al. [41]	3.93 3.95 3.22	0.90 0.85 1.03
Satisfaction	[SAT1] I am satisfied with the experience of using Gather Town as a Virtual Space [SAT2] My decision to interact through Gather Town was the right one [SAT3] I am happy to use Gather Town for my video calls [SAT4] I am happy to interact with other people through Gather Town	Ho and See-To [20] Palos-Sanchez et al. [38] Katz et al. [22] Rauschnabel et al. [41]	3.16	0.86
			3.27	0.91
			2.90	1.03
			3.40	0.95

About the items, we can affirm that the items that value the tool the most are [FUN1], [ENT1] and [ENT2] with values between 3.80, 3.93 and 3.95 respectively. This indicates that participants rate as practically very close to “agree” the calculated means of Interaction and learning with peers in non-face-to-face classroom activities would be enhanced by using applications such as Gather Town, Gather Town could be entertaining for me, and Gather Town looks like fun to use.

Participants rate [SAT3] and [SAT1] lower with values of 2.90 and 3.16 respectively. This means that they rate indifferently items such as I am happy to use Gather Town for my video call or I am satisfied with the experience of using Gather Town as a Virtual Space.

Regarding the calculated standard deviation, the values where there is the highest degree of consensus are those related to [SAT3] I am happy to use Gather Town for my video calls.

## Discussion

The lockdown that took place in 2020 sped up the adoption of telework by companies to an extent that was difficult to predict. Though teleworking as such might not be new, the challenges of managing remote teams certainly are for companies that only worked remotely one day a week, or when they needed to do so.

Despite with the rapid development of communications and computer networks, big data, smart cities, etc. have emerged [51], unfortunately, some challenges do not have a straightforward solution, as managing teams remotely is simply not the same as doing so face-to-face, which means that applying the same strategies that are used offline, does not always give way to the same benefits when working remotely.

This research aims to help with one of such conundrums, in this case, by exploring employees’ thoughts on the use of virtual worlds to boost cohesion within teams. A short answer to this question based on the results presented in the last section would be that virtual worlds could bring something to the table. But things are not that simple, so the results have been broken down in the following sections so that the reader form their own opinion on whether if, or not, Gather Town and other similar virtual worlds would be the best solution.

### Would gather town be a practical tool to use?

This information was gathered through the questions about teams being united. As was presented in the results section, employees from all groups defined cohesive teams in terms of quality of communication within the team, coordination, and having a common objective. This is hardly surprising as these are important constructs that have been extensively examined in virtual team literature. According to Garro-Abarca et al. [16], for example, the perception of cohesion would be based on the communication within the team, so it is hardly a surprise that communication came up so many times as a way for employees to measure how united their teams are. Being coordinated could be considered a consequence of having good communication in a team, and having common goals could be related to an important antecedent of cohesion listed [14], which would be task commitment (defined by the author as the strength of the group’s focus on goal attainment).

Other elements that were highlighted by fewer participants, but not less important, were trust and empathy. They felt trust was very important to be able to communicate correctly, and that being aware of colleagues’ feelings and being able to offer help, as well as receive it, was very important. This is probably why, many felt that some of the most important things that had been lost in the transition from face-to-face workplaces to remote modalities, was the physical access to their colleagues, which meant they also lost a lot of non-verbal information, as well as the readiness of having someone physically available.

Additionally, many participants mentioned that working as a group, and not as individuals were also tremendously important. In this sense that if something went wrong, all of the group would take the hit for it, and if there was a victory to celebrate, the same would happen. This element could be linked to how participants view themselves in terms of their social identity, in line with [48] initial theory. Members considering themselves as part of a group would be another of the antecedents [14] would consider for group cohesion.

### What has physical distance taken away employees those virtual worlds could give back?

When participants stopped being able to meet at the office, the main thing they would have lost would be a great deal of information. It is probably for this reason that most of their comments regarding the effects of physical distance are on how they have had less feedback on how other people felt, lower quality communication, more difficulty sharing information, getting help, and learning how to do new tasks. They also commented on a greater wastage of time because they felt they had lost the ability to consult with their colleagues directly.

Virtual worlds have the advantage, or disadvantage, that users are represented by avatars in a virtual space, as is the case with other activities in which technology is a fundamental element [50]. Even an avatar, although not in such a straightforward manner, conveys information about its user. Additionally in Gather Town, users have the option of turning on their cameras. Many employees agreed on the fact that Gather Town did offer more context information than other tools that were currently being used because it showed what colleagues were doing at each time, although some adjustments would have to be made.

### What are employees’ views on gather town as a tool to boost task and social cohesion?

Most participants felt like Gather Town’s main benefit would be in boosting social cohesion. Some of them were able to come up with a few scenarios where it could be used for specific tasks, but most of them did not think it would be compatible with their work and did not know how it could be integrated into their daily work life or did not feel comfortable with the idea of being online all the time, which would be necessary if they wanted to provide this additional information.

However, they were able to think of some situations where it could be useful. For example, some of the developers thought said that if they found a way to share the code that they otherwise would share via Google Meets, it could be done through Gather as well. It was also suggested that it could be used for stand-alone events, such as when one of the bosses needed to communicate something to the whole company.

Another interesting line of discussion started in group 4, where they commented it could be used for training and team-building activities. In this group, one of the members as part of the human resource department, and it was interesting to see how from her point of view, this would be considered a task cohesion activity, while the rest of the group thought of it as social.

Most participants thought it would be ideal for improving social cohesion. Some of the reasons behind this were that they felt it was fun, entertaining, and a good environment to socialize. As previously mentioned, one of the participants said using it had helped her wind down when playing around with the objects in the room.

## Conclusions

Employees were able to use Gather Town without any major trouble. Although they considered that some of the tools that were available to create new rooms could be a bit complex to handle at first, they were all successful at completing the task of creating their virtual office.

Something most of them felt very strongly about was how communication in a face-to-face environment is preferable (this does not mean that they dislike working remotely). One of the things many of them appreciated about non-virtual settings was the casual interactions they were able to have with their colleagues at the office. Interestingly, one of the things they appreciated about Gather Town was its capacity to give additional information on what their colleagues were doing at the time, and its potential to recreate these same casual interactions.

Based on their answers, it looks like they would be ready to use virtual worlds from time to time for specific activities, and they believe they would add more value to the social side of their work rather than the technical one. Although they did consider Gather Town could be particularly interesting for workers that had to spend long periods online together.

Regarding cohesion, which is the core of this investigation, employees felt that for a group to be united, that is, for there to be cohesion within a group, it would have to have good communication, there would have to be trusted among its members, and it would be key for them all to row in the same direction.

In this sense, they were able to identify several characteristics Gather Town has that could help with improving these factors. A few have been mentioned in the paragraphs above (casual interaction, giving others additional information on what someone is doing, which would help with knowing how to communicate with them without non-virtual cues). They also felt it was important that users had their cameras on, and that Gather, or any other tool they used for communicating, could allow for informal communication to exist. They felt that e-mails, in contrast, created a barrier that made deep communication more complicated because they tended towards more formal language.

Finally, employees considered that whatever tools were used it was important to set routines (online coffee sessions and so on) and to make a conscious effort to actually “keep cohesiveness” in the group. In this sense, we believe Gather Town and similar tools could have a lot of potentials since they allow for different kinds of events compared to traditional video tools.

When asked about how they felt about Gather and task and social cohesion, they seemed to think it could be better used to improve relationships between them, perhaps for the reasons we mentioned earlier. It is interesting however to observe that people from very different departments (finance, HR, sales, programming…) were able to collaborate easily using a virtual world and coordinate their efforts to reach a common goal. Perhaps both types of cohesion could be benefitted from virtual worlds more than they thought.

### Limitations and future directions

Because this was a qualitative study, and only a company employees participated in it, we believe it is a good starting point to investigate how virtual settings could help teams that work remotely become more cohesive. The results are therefore limited to the experiences of participants within the company, as well as not being statistically significant in any way. Furthermore, because both focus group moderation and analysis were performed by the same person, future qualitative investigations would benefit from having a larger and more experienced team on board.

Future research could expand on these results in various ways. Further qualitative studies could look at other sectors, research other virtual worlds other than Gather Town and explore the differences between them.

Quantitative studies could also be designed to measure how and when virtual worlds increase cohesion, perhaps through communication, as it was one of the main factors employees identified to be fundamental for their own teams to be united.

Furthermore, because this study was limited to a one-time exposition of the tool, it would be essential to design a longitudinal study to help us observe how cohesion within teams’ changes over time.

Lastly, future research could also investigate immersive technologies and their relationship with cohesion, since it does look like virtual worlds will, shortly, be immersive as well.

## Declarations

### Data availability

The datasets used and analyzed during the present study are available, properly anonymized, from the corresponding author on reasonable request.
